# Chemical shift assignments of calmodulin bound to a C-terminal site (residues 1120–1147) in the β-subunit of a retinal cyclic nucleotide-gated channel (CNGB1)

**DOI:** 10.1007/s12104-022-10101-7

**Published:** 2022-08-20

**Authors:** Aritra Bej, James B. Ames

**Affiliations:** Department of Chemistry, University of California, Davis, CA 95616 USA

**Keywords:** CaM, Calcium, CNGB1, Retina, Photoreceptor, NMR

## Abstract

Retinal cyclic nucleotide-gated (CNG) channels consist of two protein subunits (CNGA1 and CNGB1). Calmodulin (CaM) binds to two separate sites within the cytosolic region of CNGB1: CaM binding to an N-terminal site (human CNGB1 residues 565–587, called CaM1) decreases the open probability of CNG channels at elevated Ca^2+^ levels in dark-adapted photoreceptors, whereas CaM binding to a separate C-terminal site (CNGB1 residues 1120–1147, called CaM2) may increase channel open probability in light activated photoreceptors. We recently reported NMR chemical shift assignments of Ca^2+^-saturated CaM bound to the CaM1 site of CNGB1 (BMRB no. 51222). Here, we report complete NMR chemical shift assignments of Ca^2+^-saturated CaM bound to the C-terminal CaM2 site of CNGB1 (BMRB no. 51447).

## Biological context

Retinal CNG channels in rod photoreceptors conduct a cation current in response to light-dependent changes in intracellular levels of cGMP that occur during visual phototransduction (Baylor 1996; Fesenko et al. 1985). CaM binding to retinal CNG channels mediates Ca^2+^-dependent modulation of channel open probability, which may contribute to light adaptation in retinal rod cells (Bradley et al. 2005; Fain et al. 2001; Hsu and Molday 1993). Retinal CNG channels consist of two protein subunits, CNGA1 and CNGB1 (Bradley et al. 2001). The CNGA1 subunit forms a functional homo-tetrameric channel in the absence of CNGB1, whereas CNGB1 does not form a functional homomeric channel (Finn et al. 1998). In native rod cells, CNG channels form a hetero-tetramer that consists of 3 CNGA1 bound to 1 CNGB1 in a Ca^2+^-dependent fashion (Shuart et al. 2011). A recent cryoEM structure of the retinal CNG channel (Barret et al. 2021) revealed that a C-terminal site in CNGB1 called CaM2 (residues 1120–1147) is bound to the C-terminal domain of CaM (residues 80–149, called C-lobe). However, the cryoEM image lacked sufficient resolution to discern atomic-level structural interactions between CaM and CNGB1, and the structure of the CaM N-lobe in the complex was completely missing. CaM was also suggested to bind to a separate N-terminal site in CNGB1 called CaM1 (residues 565–589) (Trudeau and Zagotta 2002) that may regulate CNGB1 binding to CNGA1 (Shuart et al. 2011) and perhaps mediate Ca^2+^-induced CNG channel inactivation in rod cells (Hsu and Molday 1993; Trudeau and Zagotta 2003). Defects in the Ca^2+^-dependent regulation of CNG channels are genetically linked to autosomal recessive retinitis pigmentosa and other inherited forms of blindness (Bareil et al. 2001). Elucidating the Ca^2+^-dependent CNG channel interaction with CaM bound at two separate sites may provide insights for the treatment of retinal diseases. We report here NMR resonance assignments of Ca^2+^-saturated CaM bound to the CaM2 site of CNGB1 (hereafter called CaM/CaM2). These assignments are an important step toward elucidating the complete structure of CaM bound to CNGB1.

## Methods and experiments

### Expression and purification of CaM

Human CaM was overexpressed in *E. coli* strain BL21(DE3) using pET11b (Novagen) and the expressed protein was purified as described previously (Bej and Ames 2022a). The purity of the protein samples was confirmed by sodium dodecyl sulfate–polyacrylamide gel electrophoresis (SDS-PAGE). The CaM2 peptide (CNGB1 residues 1122–1143) was purchased from GenScript. The CaM2 peptide was added in threefold excess to Ca^2+^-bound CaM and concentrated to 0.4 mM in a final volume of 0.3 ml.

### NMR spectroscopy

**Fig. 1:**
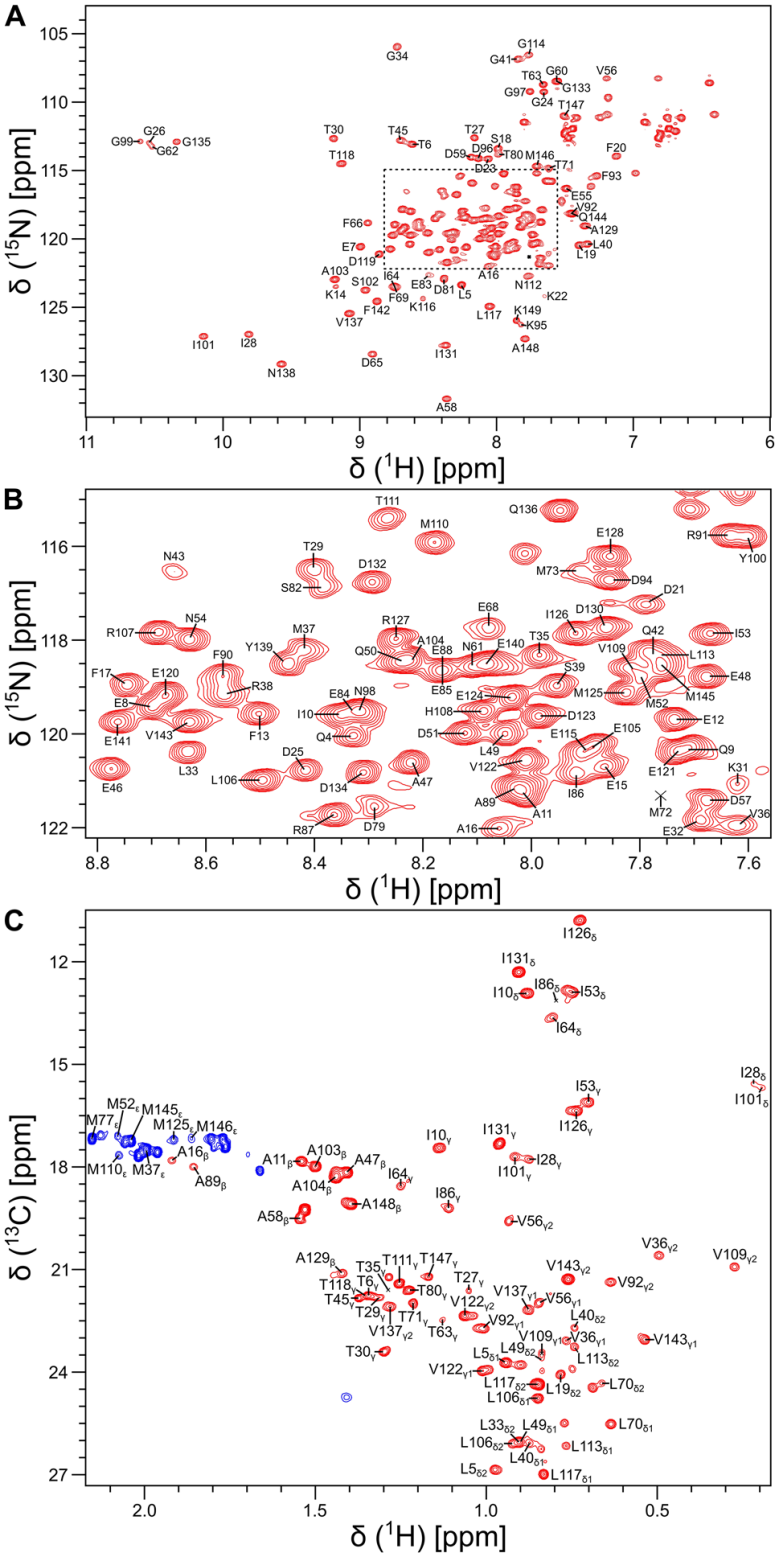
NMR spectroscopy of isotopically labeled CaM bound to unlabeled CaM2 peptide. **A** ^15^N-^1^H HSQC spectrum of CaM/CaM2 recorded at 800 MHz ^1^H frequency. **B** Close-up view of resonance assignments in the spectrally crowded region. **C** Constant-time ^13^C-^1^H HSQC spectrum of CaM/CaM2. Complete resonance assignments are available at BMRB 51447

All NMR samples of isotopically labeled CaM bound to the unlabeled CaM2 peptide (called CaM/CaM2) were prepared in 20 mM Tris-d_11_ (pH 7.0) and 1 mM CaCl_2_ containing either 8% or 100% (v/v) D_2_O and placed in a Shigemi NMR tube (Shigemi Inc.). NMR experiments (at 308 K) were performed on a Bruker Avance III 800 MHz spectrometer equipped with a triple resonance cryogenic (TCI) probe. The ^15^ N-^1^H HSQC spectrum (Fig. 1A, B) contained 200 × 2048 complex points for ^15^N(F1) and ^1^H(F2). Backbone resonances were assigned by analyzing triple resonance spectra: HNCACB, CBCA(CO)NH, HNCO, HBHA(CO)NH, and HBHANH. Side chain resonances (aliphatic (Fig. 1C) and aromatic) were assigned by analyzing HCCCONH-TOCSY, HCCH-TOCSY, HBCBCGCDHD and HBCBCGCDCEHE as described previously (Ikura et al. 1991). The NMR data were processed using NMRPipe (Delaglio et al. 1995) and assignment was performed using Sparky (Lee et al. 2015).

## Extent of assignments and data deposition

Backbone resonance assignments of CaM/CaM2 are illustrated by labeled peaks in the ^15^N-^1^H HSQC spectrum of CaM/CaM2 (Fig. 1A, B). Side chain methyl resonance assignments are illustrated by the labeled peaks in the constant-time ^13^C-^1^H HSQC spectrum (Fig. 1C). NMR assignments were derived from 3D heteronuclear NMR experiments performed on ^13^C/^15^N-labeled CaM bound to unlabeled CaM2 peptide. The high degree of chemical shift dispersion and uniform peak intensities indicate that CaM/CaM2 complex is stably folded. The large downfield chemical shifts of the amide resonances assigned to G26, G62, G99 and G135 indicate that Ca^2+^ is bound to each of the four EF-hands (Fig. 1A). The large upfield chemical shifts of methyl resonances assigned to residues I28, V36, I101 and V109 (Fig. 1C) suggest that these residues may be located in the hydrophobic core near aromatic residues. The NMR linewidth of the V109 resonance for CaM/CaM2 is much sharper than it is for CaM/CaM1 (Bej and Ames 2022a), suggesting that the CaM C-lobe binds to CaM2 with higher affinity than it binds to CaM1. At least 92% of the backbone resonances (^1^HN, ^15^N, ^13^Cα, ^13^Cβ, and ^13^CO) and 85% of side-chain resonances were assigned. Three residues in the second EF-hand of CaM (A74, R75, and K76) could not be assigned, because their HSQC peaks could not be detected. These same resonances are exchange broadened in CaM bound to the CNGB1 CaM1 peptide (Bej and Ames 2022a) and the α-subunit of the retinal cyclic nucleotide-gated channel (CNGA2) (Contessa et al. 2005), but are not exchange broadened in free CaM (Bej and Ames 2022b; Kainosho et al. 2006). The chemical shift assignments (^1^H, ^15^N, ^13^C) for CaM/CaM2 have been deposited in the BioMagResBank (http://www.bmrb.wisc.edu) under accession number 51447.

**Fig. 2:**
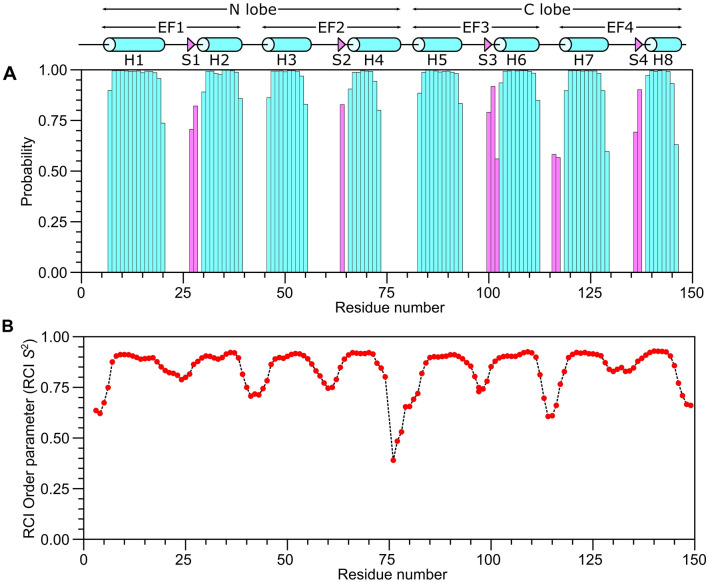
Secondary structure and order parameters of CaM/CaM2. **A** Secondary structure probability and **B** RCI order parameter (RCI-S^2^) were predicted using TALOS + (Shen et al. 2009). Secondary structural elements are depicted by cylinders (helix) and triangles (strand) derived from the CaM crystal structure (PDB ID—2VAY (Halling et al. 2009))

**Fig. 3:**
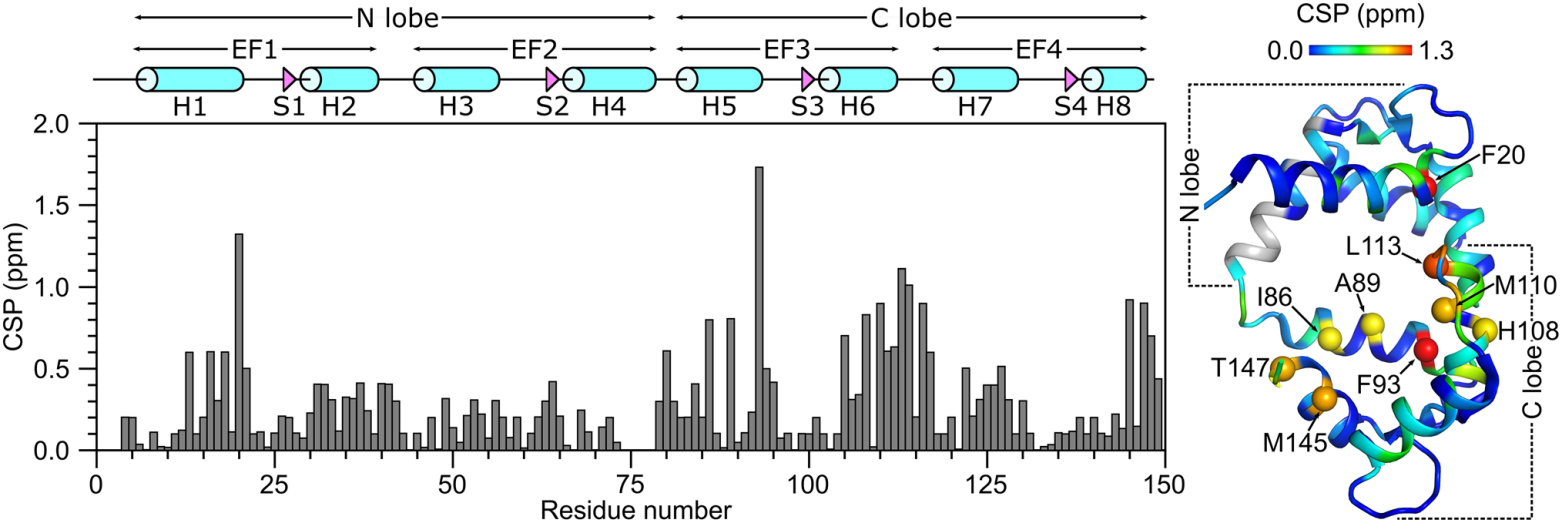
Chemical shift perturbation (CSP) versus residue number for CaM/CaM2. CSP was calculated as: $CSP= \sqrt{{\left(\Delta {H}^{N}\right)}^{2}+{\left(\Delta N\right)}^{2}}$, where ΔH^N^ and ΔN are the difference in the ^1^H^N^ and ^15^N chemical shifts, respectively for CaM/CaM2 versus free CaM in the absence of CaM2 (Bej and Ames 2022b). CSP values are superimposed on the CaM crystal structure (PDB ID: 2VAY (Halling et al. 2009))

The secondary structure of CaM/CaM2 was calculated on the basis of chemical shift index (Wishart et al. 1992) and ANN-Secondary structure prediction using TALOS + (Shen et al. 2009) (Fig. 2). The secondary structure of CaM/CaM2 is identical to that reported previously for CaM/CaM1 (Bej and Ames 2022a), and is depicted by cylinders and triangles in Fig. 2A. A preliminary analysis of NOESY-derived distances indicate that the eight α-helices and four β-strands combine to form four EF-hands (EF1: residues 7–39, EF2: residues 45–76, EF3: residues 83–112 and EF4: residues 119–144) as seen in the crystal structure of Ca^2+^-bound CaM (Babu et al. 1988). The two N-terminal EF-hands (EF1 and EF2) interact with one another to form the CaM N-lobe, and the two C-terminal EF-hands (EF3 and EF4) form the C-lobe. The CaM2 peptide binds to CaM and causes chemical shift perturbations (CSPs) observed for CaM residues in both the N-lobe and C-lobe (Fig. 3), suggesting that the CaM2 peptide is making contact with both lobes of CaM as seen in previous structures of CaM bound to other peptides (Hoeflich and Ikura 2002). The CSP values for the CaM/CaM2 complex in this study are somewhat higher on average than the CSP values observed previously for CaM/CaM1 (Bej and Ames 2022a). The higher CSPs caused by CaM2 binding might be explained by higher affinity CaM binding to CaM2 compared to CaM1. The CSP values for C-lobe residues (I86, A89, F93, H108, M110, L113, M145 and T147) are detectably higher than the CSPs for the corresponding residues in the N-lobe. However, N-lobe residues F13, A16 and F20 have relatively high CSP values (above 0.5) that suggest these residues may be contacting the CaM2 peptide. On average, the C-lobe has higher CSP values than the N-lobe, which suggests that CaM2 may bind to the CaM C-lobe with higher affinity than that of the N-lobe. This is in stark contrast to CaM binding to the N-terminal CNGB1 peptide (CaM1) in which the CaM N-lobe exhibits higher CSP values (Bej and Ames 2022a). On the basis of our CSP analysis, we suggest that a single CaM may bind to CNGB1 in which the CaM C-lobe preferentially binds to the C-terminal CNGB1 site (CaM2) and the CaM N-lobe preferentially binds to the N-terminal CNGB1 site, CaM1. Future studies are needed to test this possibility by measuring the binding stoichiometry of CaM bound to the full-length CNG channel. The NMR assignments of CaM/CaM2 presented here are an important first step toward determining the full three-dimensional structure of CaM bound to CaM2.

## Data Availability

The assignments have been deposited to the BMRB under the accession code: 51447.
